# Relevance of anti‐thyroperoxidase antibodies in immune thrombocytopenia in adults: A single‐centre cohort study

**DOI:** 10.1111/bjh.70745

**Published:** 2026-08-12

**Authors:** Nicolas Duhamel, Marie Ottavi, Sarah Lechtman, Nihal Martis, Charlotte Hinault‐Boyer, Julie Merindol, Pierre‐Yves Jeandel, Michael Levraut

**Affiliations:** ^1^ Service de Médecine Interne et Immunologie Clinique Centre Hospitalier Universitaire de Nice Nice France; ^2^ Laboratoire d'hormonologie Centre Hospitalier Universitaire de Nice Nice France; ^3^ URRIS, Unité de Recherche Clinique Côte d'Azur Centre Hospitalier Universitaire de Nice Nice France

**Keywords:** anti‐thyroperoxidase antibodies, haemorrhagic syndrome, immune thrombocytopenia

To the Editor,

Immune thrombocytopenia (ITP) is an acquired autoimmune disorder causing potentially severe bleeding with isolated thrombocytopenia (platelet count <100 × 10^9^/L). Although treatment strategies are well established, biomarkers to predict first‐line treatment response, relapse and long‐term outcomes are lacking. Screening for anti‐nuclear (ANA), anti‐phospholipid (APL) or anti‐thyroid autoantibodies (ATA) is suggested during the diagnostic process.^1^, ^2^ ANA are detected in up to 20%–50% of patients with primary ITP^3^, ^4^ and may indicate a higher risk for chronic disease and overexposure to second‐line therapies. ATA, particularly anti‐thyroperoxidase (anti‐TPO) antibodies, are found in 10%–30% of ITP patients.^5^ Anti‐TPO antibodies are associated with other autoimmune diseases such as Hashimoto's thyroiditis and recurrent miscarriage. Their role in ITP remains unclear. In children, ATA positivity correlates with lower platelet counts at diagnosis but not with treatment response or disease duration.^6^ Given the lack of data regarding the role of ATA in adults with ITP, we conducted a retrospective cohort study to evaluate the prognostic value of anti‐TPO in adults with ITP.

Patients followed at the Department of Internal Medicine, University Hospital of Nice, France, aged 18 years or older, diagnosed with ITP,^2^ having anti‐TPO antibody measurement at diagnosis and ≥1 year follow‐up were included. Demographic, clinical and biological data were collected at the time of ITP diagnosis and during follow‐up. Anti‐TPO antibodies were measured using the chemiluminescence analyser Atellica SII (Siemens, healthineers). The presence of anti‐TPO antibodies was set when the value was over 60 mUI/L (Limit of Quantification [LOQ] < 28 mUI/L; Variation Coefficient [VC] < 5%). The study was approved by the Institutional Review Board of Nice University Hospital (IRB number 2025‐710).

Secondary ITP was defined as ITP associated with prevalent or concomitant chronic infection, autoimmune disease, haematological malignancy or primary immunodeficiency. Other patients were diagnosed with primary ITP. Severe bleeding was defined as a bleeding score >8.^7^ An overall response (OR) was defined as a platelet count >30 × 10^9^/L and at least twice baseline value, without bleeding. A complete response (CR) was defined as an OR with platelet >100 × 10^9^/L. A sustained response (SR) was defined as an OR lasting for 6 months without treatment change. Chronic ITP was diagnosed in patients with a platelet count of <100 × 10^9^/L or who required second‐line treatment to maintain platelet above this level for more than 12 months.

The association between baseline covariates and anti‐TPO antibodies status was analysed using chi‐squared or Mann–Whitney test as appropriate. Spearman's coefficient was used for correlation analysis. To evaluate whether anti‐TPO antibodies were independently associated with the bleeding risk score at diagnosis or at first relapse, anti‐TPO‐positive and ‐negative ITP patients were matched 1:1 using propensity score matching without replacement. Propensity scores were calculated using logistic regression model including the following normalized covariates: anti‐thrombotic drugs use, the presence of an associated disorder (primary vs. secondary ITP) and age. All analyses were two‐tailed and *p*‐values less than 0.05 were considered statistically significant. Analyses were performed with EasyMedStat version 3.40.

Of 164 adults with ITP screened, 109 were excluded for missing baseline anti‐TPO measurement or follow‐up less than 12 months and 55 included. Twenty‐seven of 55 were female, with a median age at diagnosis of 49 (29; 65) years. Primary ITP was frequent (*n* = 42 [76%]). Patients with secondary ITP were well balanced and represented 23% (*n* = 6) in the anti‐TPO‐positive group and 24% (*n* = 7) in the anti‐TPO‐negative group (Table 1). In anti‐TPO‐positive patients, only four had thyroid dysfunction and none of them hyperthyroidism. Anti‐TPO‐positive patients were older (*p* = 0.032) and presented with more severe disease, characterized by significantly lower platelet counts (*p* = 0.014) and higher median bleeding score (*p* < 0.001). Anti‐TPO‐positive patients were more likely to take anti‐thrombotic drugs (*p* = 0.008) and to require first‐line therapy (*p* = 0.044), particularly the use of intravenous immunoglobulins (79% vs. 38%, *p* = 0.007), related to greater bleeding score. All data are provided in Table 1.

**TABLE 1:** Cohort description at ITP diagnosis according to anti‐TPO antibody status.

	TPO positive	TPO negative	*p*‐value
	*n* = 26	*n* = 29	
Median age, years (IQR)	59 (45; 71)	37 (23;64)	**0.032**
Female gender, *n* (%)	15 (58)	12 (41)	0.348
Mean anti‐TPO titre (UI/mL, min‐max)	935 (122–5000)	25 (0–55)	**<0.001**
Hypothyroidism, *n* (%)	4 (15)	1 (3)	0.178
Median bleeding score (IQR)	9 (6; 11)	2 (0; 5)	**<0.001**
Patients with severe bleeding, *n* (%)	15 (62)	3 (10)	**<0.001**
Mucosal	13	2	
ENT	5	0	
Gastrointestinal	2	1	
Urinary tract	1	0	
Brain	0	0	
Median platelets count, G/L (IQR)	4 (2; 12)	10 (5; 53)	**0.014**
Anti‐thrombotic drug use, *n* (%)	10 (38)	2 (7)	**0.008**
Anti‐platelets agent, *n* (%)	6 (23)	2 (7)	0.131
Anti‐coagulants, *n* (%)	4 (15)	0 (0)	**0.044**
Secondary ITP, *n* (%)	6 (23)	7 (24)	0.999
Median follow‐up duration (months) (IQR)	23 (15; 37)	23 (10; 41)	0.794
Chronic ITP	14 (54)	19 (66)	0.663
Immunobiology			
Positive ANA, *n* (%)	13 (50)	8 (29)	0.182
Positive lupus anti‐coagulant, *n* (%)	2 (11)	7 (28)	0.263
Positive ELISA APL antibody, *n* (%)	0 (0)	2 (8)	0.467
First‐line treatment	24 (92)	20 (69)	**0.044**
Steroids, *n* (%)	23 (96)	19 (95)	0.999
IVIg, *n* (%)	19 (79)	8 (38)	**0.007**
Other drugs, *n* (%)	3 (12)	0 (0)	0.236
Overall response to first‐line treatment, *n* (%)	24 (100)	20 (100)	0.999
Complete response to first‐line treatment, *n* (%)	20 (83)	12 (63)	0.170
First‐line treatment response delay (days) (IQR)	4 (3; 7)	3 (2; 6)	0.228
Sustained response, *n* (%)	10 (56)	9 (47)	0.746
Relapse, *n* (%)	12 (50)	17 (68)	0.322

*Note*: Statistical significance was defined as *p*‐value < 0.05.

Abbreviations: ANA, antinuclear antibody; APL, antiphospholipid antibody; ELISA, Enzyme‐Linked Immunosorbent Assay; ENT, ear nose and throat; IQR, interquartile range; ITP, immune thrombocytopenia; IVIg, intravenous immunoglobulins; TPO, thyroperoxydase antibody.

In the entire cohort, the bleeding risk score at the time of ITP diagnosis was associated with the use of anti‐thrombotic drugs (median score of 8 [4; 10] vs. 3 [0; 7], *p* = 0.049), age (*ρ* = 0.43; *r* ^2^ = 0.175; *p* = 0.001) and anti‐TPO antibody titre (*ρ* = 0.34; *r* ^2^ = 0.025; *p* = 0.013). The association between the bleeding risk score and anti‐TPO antibody status or titres remained significant when analysing patients with primary ITP (median bleeding score of 9 [6; 11] vs. 2 [0; 5], *p* < 0.001 and *ρ* = 0.39; *r* ^2^ = 0.032; *p* = 0.009 respectively). After matching patients for age, the use of anti‐thrombotic drugs and primary versus secondary ITP status, anti‐TPO‐positive patients still presented with a higher median bleeding score (9 [6; 10] vs. 3 [0; 6], *p* = 0.013) and more frequent severe bleeding (10 [63%] vs. 2 [11%], *p* = 0.003) (Table 2).

**TABLE 2:** Bleeding score at ITP diagnosis and first relapse according to anti‐TPO antibody status after matching patients for age, the use of anti‐thrombotic drugs and ITP‐associated disorders.

	TPO positive	TPO negative	SMD	*p*‐value
	*n* = 18	*n* = 18		
Median age, years (IQR)	52 (43; 67)	55 (30; 73)	0.0003	0.999
Anti‐thrombotic drug use, *n* (%)	2 (11)	2 (11)	0	0.999
Anti‐platelets agent, *n* (%)	2 (11)	2 (11)		
Anti‐coagulants, *n* (%)	0 (0)	0 (0)		
Secondary ITP, *n* (%)	3 (17)	3 (17)	0	0.999
Median bleeding score (IQR)	9 (6; 10)	3 (0; 6)	—	**0.013**
Patients with severe bleeding, *n* (%)	10 (63)	2 (11)	—	**0.003**
Median platelets count, G/L (IQR)	5 (2; 12)	7 (3; 36)	—	0.340
Patients with relapse, *n* (%)	10 (59)	10 (59)	0	0.999
Median time to first relapse (months) (IQR)	1.8 (1.1; 3.7)	6.5 (1.8; 10.4)	—	0.186
Median bleeding score at first relapse (IQR)	3 (2; 7)	0 (0; 2)	—	**0.028**
Patients with severe bleeding at first relapse, *n* (%)	3 (30)	1 (10)	—	0.582

*Note*: Statistical significance was defined as *p*‐value < 0.05.

Abbreviations: IQR, interquartile range; ITP, immune thrombocytopenia; SMD, standardized mean differences; TPO, thyroperoxydase.

From the 29 patients who experienced a relapse, the median bleeding score at first relapse was higher in the anti‐TPO‐positive group (3.5 vs. 0, *p* = 0.005—Table S1). From the matched cohort (*n* = 18 patients in each group), 10 patients in each group experienced a first relapse. Anti‐TPO‐positive patients had a higher median bleeding score at first relapse than anti‐TPO‐negative patients (median bleeding score of 3 [2; 7] vs. 0 [0; 2], *p* = 0.028). Three patients in the anti‐TPO‐positive group (30%) and one patient in the anti‐TPO‐negative group (10%) experienced severe bleeding; the latter shifted from anti‐TPO negative to anti‐TPO positive (Table 2).

There was no difference in OR and CR to first‐line treatment according to anti‐TPO status. The median time to OR was 4 (1; 7) days in the anti‐TPO‐positive group and 3 (2; 6) days in the anti‐TPO‐negative group (*p* = 0.228). We found similar rates of SR after first‐line treatment, relapse, exposure to second‐line therapy and chronic ITP in both groups.

Our study confirmed a high prevalence of immunological markers in adults with ITP, as 47% had anti‐TPO, 38% had ANA and 20% had APL antibodies. Anti‐TPO antibodies‐positive patients were more likely to experience severe bleeding, low platelet counts and greater exposure to first‐line treatment at diagnosis, including intravenous immunoglobulins, independently of age and the use of anti‐thrombotic drugs, both known as bleeding risk factors.^8^, ^9^ Interestingly, anti‐TPO positivity at diagnosis correlated with higher bleeding scores at first relapse, and all patients with severe relapse were anti‐TPO positive. Therefore, anti‐TPO antibodies may serve as a severity biomarker in adult patients with ITP. Our results align with paediatric data showing that ATA positivity was associated with lower platelet count at diagnosis.^6^ We found no association between anti‐TPO status and the risk of relapse, the risk chronic ITP course or the need for second‐line treatment. Our results may be conflicting with others, as Hollenhorst et al. reported an inverse relationship between anti‐TPO status and remission at last follow‐up,^10^ suggesting that anti‐TPO may predict chronic ITP. Although anti‐TPO antibodies may serve as a marker of severity, they should not guide the early management of patients with newly diagnosed ITP as we do not find any difference between both groups in response to first‐line treatment.

Numerous case reports describe immune‐mediated thyroid disorders associated with ITP, suggesting a causal relationship. However, exact role of ATA during ITP remains unknown. We hypothesize that the presence of anti‐TPO antibodies, even without a confirmed Hashimoto's disease, may reflect a trend to greater autoimmunity and an associated B cells dysregulation. B‐cell activation may be associated with more anti‐platelet or anti‐thrombopoietin antibodies secretion, both responsible for accelerating platelet destruction.^11^ Since the 1950s, humoral immunity has been associated with ITP physiopathology^12^ as platelets antigens (such as Glycoprotein [Gp]IIb‐IIIa and GPIb‐IX) trigger platelet phagocytosis and complement‐mediated lysis. Additional mechanisms responsible for thrombocytopenia include impaired megakaryopoiesis and T‐cell activation.^13^ Since non‐response to rituximab has been associated with Cluster of Differentiation (CD)8+ T‐cell activation,^14^ we hypothesized that each patient or group of patient display may tend towards a more ‘B‐cell’ or ‘T‐cell’ phenotype.

It is well established that platelet counts below 20 × 10^9^/L are associated with increased severity and risk of life‐threatening bleeding.^15^ Many studies found a correlation between thyroid dysfunction and coagulation disorders, but results are conflicting as it is unclear whether coagulation disorders are related to thyroid hormones levels or the presence of ATA. Unfortunately, to our knowledge, there is no evidence of a direct interaction between anti‐TPO antibodies and endothelial cells, platelets or coagulation factors.

Our study has important limitations. Due to its retrospective design, our results may suffer from interpretation bias. In addition, most of the patients screened in the study did not have anti‐TPO measurements at baseline. However, as it is not recommended as a routine investigation, the inconsistent availability of anti‐TPO measurements likely reflects differences in individual clinical practice rather than a systematic selection of patients based on thyroid‐related characteristics. The small number of patients analysed may have influenced our conclusions. Nevertheless, our results are consistent with a recent paediatric study, which strengthens their scientific value. Our study included both primary and secondary ITP, and the underlying cause of ITP may have influenced the response to first‐and second‐line therapies. Nonetheless, both primary and secondary ITP were well balanced between both positive and negative TPO antibodies groups and our analysis was performed in adjusting for such variable. Therefore, it may have influenced our conclusions.

In conclusion, we encourage the measurement of anti‐TPO antibodies as a non‐invasive and inexpensive test at the time of ITP diagnosis, which may provide interesting data for the risk of severe bleeding in ITP. Data from a larger cohort may clarify the role of anti‐TPO antibodies in adults with ITP.

## AUTHOR CONTRIBUTIONS

**Marie Ottavi:** Writing – review and editing; resources; visualization. **Julie Merindol:** Writing – review and editing; resources; visualization. **Nicolas Duhamel:** Conceptualization; writing – original draft; methodology; validation; writing – review and editing; investigation; resources; data curation; visualization; formal analysis. **Sarah Lechtman:** Writing – review and editing; resources; visualization. **Charlotte Hinault‐Boyer:** Writing – review and editing; resources; visualization. **Pierre‐Yves Jeandel:** Writing – review and editing; resources; supervision; visualization. **Michael Levraut:** Conceptualization; investigation; writing – original draft; writing – review and editing; validation; methodology; supervision; resources; data curation; formal analysis; visualization. **Nihal Martis:** Writing – review and editing; resources; visualization.

## FUNDING INFORMATION

This study did not receive any financial support.

## CONFLICT OF INTEREST STATEMENT

The authors declare no conflicts of interest related to this study.

## Supporting information


**Table S1.** Bleeding score at first relapse according to anti‐TPO antibody status in the all cohort.

## Data Availability

Data are available upon request from the corresponding author.
