# Supplementary material for: Relevance of anti‐thyroperoxidase antibodies in immune thrombocytopenia in adults: A single‐centre cohort study
Source: Br J Haematol. 2026 Aug 12;209(4):1941–4. doi: 10.1111/bjh.70745 (PMC13674117; doi:10.1111/bjh.70745)
Supplement: Supplementary file 1 [file BJH-209-1941-s001.docx]

Supplementary table 1. Bleeding score at first relapse according to anti-TPO antibody status in the all cohort.

|  | **Anti-TPO positive patients**  **n=12** | **Anti-TPO negative patients**  **n=17** | **P-value** |
| --- | --- | --- | --- |
| Median time to first relapse, months (IQR) | 1.8 (0.9 ;3.3) | 3.6 (1.2 ;10.5) | 0.184 |
| Median bleeding score at first relapse (IQR) | 3.5 (2 ; 8) | 0 (0 ; 2) | **0.005** |
| Patients with severe bleeding a first relapse, n (%) | 4 (33) | 2 (12) | 0.198 |

Legend : IQR : interquartile range,
